# The Effect of Dysfunctional Ubiquitin Enzymes in the Pathogenesis of Most Common Diseases

**DOI:** 10.3390/ijms21176335

**Published:** 2020-09-01

**Authors:** Gizem Celebi, Hale Kesim, Ebru Ozer, Ozlem Kutlu

**Affiliations:** 1 Faculty of Engineering and Natural Sciences, Molecular Biology, Genetics, and Bioengineering Program, Sabanci University, Istanbul 34956, Turkey; gizemcelebi@sabanciuniv.edu (G.C.); halekesim@sabanciuniv.edu (H.K.); ebruozer@sabanciuniv.edu (E.O.); 2 Sabanci University Nanotechnology Research and Application Center (SUNUM), Istanbul 34956, Turkey; 3 Center of Excellence for Functional Surfaces and Interfaces for Nano Diagnostics (EFSUN), Sabanci University, Istanbul 34956, Turkey

**Keywords:** ubiquitination, E3s, DUBs, UPS, cancer, neurodegenerative disease, immune-related diseases

## Abstract

Ubiquitination is a multi-step enzymatic process that involves the marking of a substrate protein by bonding a ubiquitin and protein for proteolytic degradation mainly via the ubiquitin–proteasome system (UPS). The process is regulated by three main types of enzymes, namely ubiquitin-activating enzymes (E1), ubiquitin-conjugating enzymes (E2), and ubiquitin ligases (E3). Under physiological conditions, ubiquitination is highly reversible reaction, and deubiquitinases or deubiquitinating enzymes (DUBs) can reverse the effect of E3 ligases by the removal of ubiquitin from substrate proteins, thus maintaining the protein quality control and homeostasis in the cell. The dysfunction or dysregulation of these multi-step reactions is closely related to pathogenic conditions; therefore, understanding the role of ubiquitination in diseases is highly valuable for therapeutic approaches. In this review, we first provide an overview of the molecular mechanism of ubiquitination and UPS; then, we attempt to summarize the most common diseases affecting the dysfunction or dysregulation of these mechanisms.

## 1. Introduction

Cellular functions are highly dependent on the precise control of a single protein abundance within cells that is regulated by the equilibration of protein translation, folding, and degradation. The ubiquitination of proteins is one of the most important post-translational modifications, in which an ubiquitin, a small (8.6 kDa) regulatory protein, is attached to a substrate protein. This mechanism maintains protein homeostasis by regulating the degradation of cellular proteins, such as short-lived or long-lived regulatory proteins and damaged or misfolded proteins, mainly via the ubiquitin–proteasome or the autophagosome–lysosomal pathway (autophagy). Moreover, ubiquitination is involved in cellular processes by coordinating the cellular localization of proteins, activating or inactivating them, and modulating protein–protein interactions [1,2,3]. These effects are mediated by the addition of either a single ubiquitin (monoubiquitination) or a chain of ubiquitin proteins (polyubiquitination) [4].

In recent years, a considerable amount of research has been focused on the understanding of molecular action of ubiquitination in signaling pathways and how alterations in this mechanism ultimately lead to the development of human diseases. In this review, we summarize current knowledge of ubiquitination types and the sequential enzymatic cascade of ubiquitination as well as the involvement of this cascade with proteasomal degradation, which is known as the ubiquitin–proteasome system (UPS). Although protein homeostasis is regulated by the extensive crosstalk between the UPS and other degradation pathways (e.g., autophagy), the only focus of this review is providing insight into the failure of accurately regulating cellular enzymatic and proteolytic processes in the most common diseases, such as cancer, neurodegenerative, and immune-related diseases. 

## 2. Ubiquitin and Ubiquitin Proteasome System (UPS)

Ubiquitin is a small, evolutionarily conserved 76 amino acid polypeptide encoded by four genes (*UBA52*, *RPS27A*, *UBB*, and *UBC*) in mammals. This protein was first identified in the 1970s as a protein of unknown function expressed in all eukaryotic cells; later on, the key features of this protein, including its C-terminal tail and the seven Lysine (Lys-K) residues, were revealed in the early 1980s [5,6,7,8]. The identification of ubiquitin–protein conjugates followed the discovery of the ubiquitination pathway, which was initially characterized as an ATP-dependent proteolytic system. ATP-dependent proteolysis factor 1 (APF-1) was found as a polypeptide that was capable of covalently binding to protein substrates in an ATP- and Mg^2+^-dependent manner [9]. Afterwards, the APF-1 protein was named as ubiquitin. 

**Figure 1:**
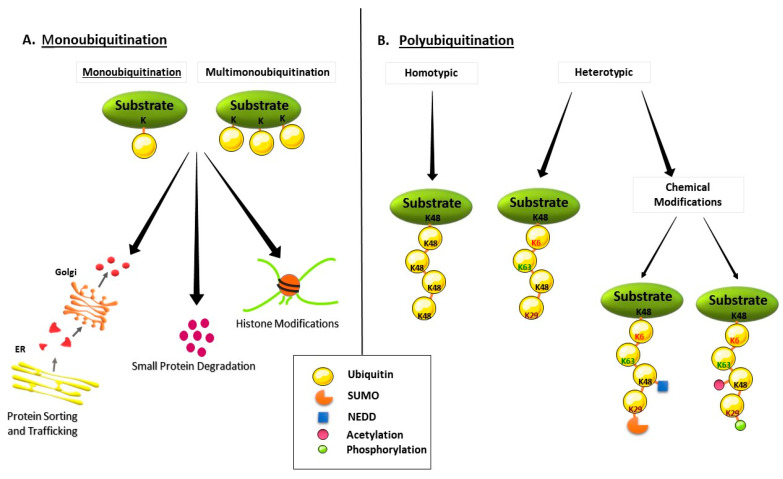
Types of Ubiquitination. (**A**) Monoubiquitination, (**B**) Polyubiquitination.

Ubiquitination is initiated by the attachment of the last amino acid (Glycine-76) of a single ubiquitin molecule to the Lys residue of one substrate protein that is called monoubiquitination. In case of the addition of one ubiquitin molecule to multiple substrate residues, the mechanism is called multi-monoubiquitination (Figure 1A). Indeed, monoubiquitination is required for the formation of a ubiquitin chain on a single Lys residue of the substrate protein, which is known as polyubiquitination. Moreover, polyubiquitin chains can be modified and turned into more complex structures by the addition of ubiquitin-like proteins (e.g., SUMO “Small Ubiquitin-like Modifier “, NEDD ”Neural Precursor Cell Expressed Developmentally Down-Regulated Protein”) or some chemical modifications (e.g., acetylation or phosphorylation) [9] (Figure 1B). In polyubiquitination, the ubiquitin protein can be ubiquitinated on its seven Lys residues (Lys6, Lys11, Lys27, Lys29, Lys33, Lys48, and Lys63) or on its N-terminus, which leads to the formation of different ubiquitin chain topologies. In fact, the ubiquitinated Lys residues as well as the ubiquitination of either the same (homotypic) or the different Lys residues (heterotypic) determine the fate of the substrate protein (Figure 1B). In other words, Lys63-linked polyubiquitin chains are associated with non-proteolytic cellular functions such as inflammation, protein trafficking, or DNA repair, while Lys48-linked polyubiquitin chains target substrates that are mostly related to the proteasomal degradation, such that the Lys48-linked ubiquitin chain can be recognized by a specific subunit of the proteasome [10].

**Figure 2:**
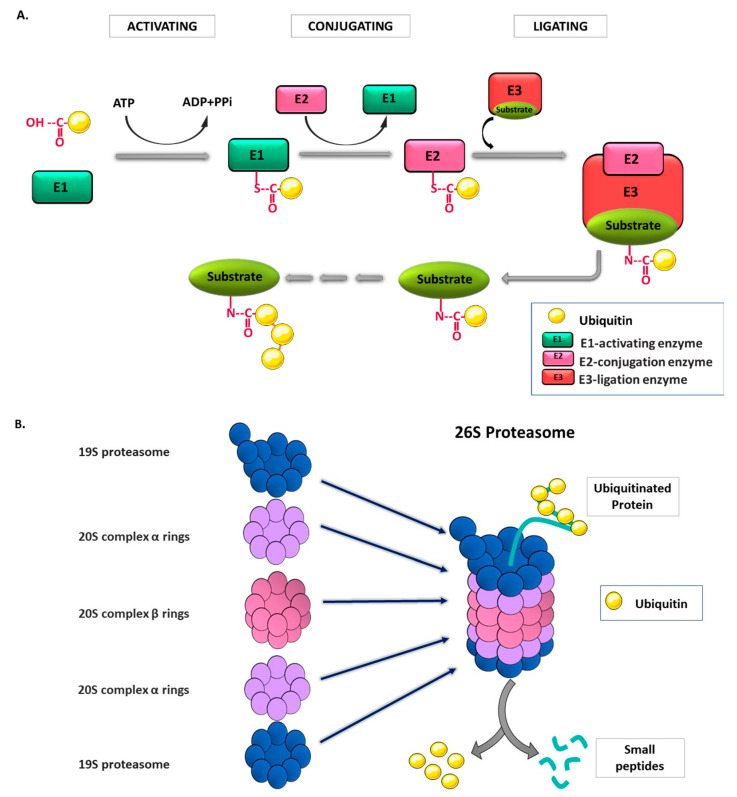
Ubiquitin proteasome system (UPS). (**A**) Enzymatic reaction of ubiquitination. For activation of the ubiquitination process, the E1 enzyme makes a thioester bond with the ubiquitin molecule, and the required energy is supplied by the hydrolysis of ATP. In the conjugation step, the ubiquitin on the E1 enzyme is transferred to the E2 enzyme. Finally, the substrate–E3 enzyme bond is involved, and the C-terminal of the ubiquitin molecule makes a covalent bond with the substrate, which is bound to the E3 enzyme. (**B**) Structure of proteasome and degradation of ubiquitinated substrates. The 26S proteasome has a multicomplex structure that is composed of a 19S regulatory units and a 20S catalytic core unit. In the 19S regulatory complex, the ubiquitinated proteins are unfolded, and ubiquitin tags are separated from the protein by deubiquitinase enzymes. Consequently, the unfolded polypeptide chain is delivered to the 20S catalytic core complex where they are degraded into small peptides.

The enzymatic mechanism of ubiquitination occurs in a proteolytic and non-proteolytic pathway consisting of three main steps: activation (E1), conjugation (E2), and ligation (E3) (Figure 2A). Initially, the binding of an E1-activating enzyme with a thioester bond activates ubiquitin in an ATP-dependent manner. Then, ubiquitin is transferred from an E1-activating enzyme to the E2-conjugation enzyme; thereafter, the E2 enzyme binds to the substrate-bound E3 ligation enzyme, resulting in a covalent attachment between the C-terminal of ubiquitin and the target substrate. As the cycle repeats, a polyubiquitination chain is formed, and the polyubiquitinated substrate is transferred to the proteasome for proteolytic degradation [11]. Considering the essential role of this enzymatic pathway in the protein homeostasis in the cell, three different enzymes (E1, E2, and E3) have been the subject of detailed research in the last decade. According to current knowledge, there are two E1 and approximately 40 E2 genes that exist in the human genome. Besides, more than 600 E3 ligase genes that are critical for the balance between ubiquitination and deubiquitination are known in the human genome [12,13]. E3 ligases are devided in three distinct groups based on their catalytic domains. The first group of E3s contains two domains: Really Interesting New Gene (RING) and UFD2 homology (U-box). The RING E3s domain coordinates Zn^2+^ binding and recruits the ubiquitin-charged E2, while the U-box E3s domain acts in a similar manner without Zn2+ coordination. The second group of E3s includes the homologous to E6–AP carboxyl terminus (HETC) domain. This domain consists of C (contains a catalytic cysteine) and N (recruits ubiquitin-charged E2) lobes that can be modulated depending on the cellular pathway. The third and last group of E3s has a RING-between-RING (RBR) motif. RING1 recruits the ubiquitin-charged E2 and transfers ubiquitin to catalytic cysteine of RING2 that conjugates ubiquitin to the substrate [14]. It is worthy of note that E3 ligases are the well-studied enzyme in this enzymatic cascade, which is most probably due to its critical role in protein quality control and homeostasis in the cell. 

The proteasomes (26S proteasome) are large and multi-catalytic proteases that are composed of a 20S barrel-shaped core complex and a 19S regulatory complex. Protein degradation occurs in the 20S complex, which consists of four stacked hollow rings, each of which has coordinately functional distinct subunits: outer α rings and inner β rings (Figure 2B). Outer α rings contain seven α subunits, and these subunits are controlled by the binding of cap structures that recognize polyubiquitin tags on the substrate proteins. β subunits contain three to seven protease active sites involved in degradation processes. Notably, the 20S proteasome has also a critical role in the dissociation of ubiquitin proteins from the substrates via deubiquitinating enzymes (DUB) [15]. Once the ubiquitinated proteins reach the 19S regulatory complex, this complex delivers them to the 20S proteasome. The 19S proteasome has 18 different subunits, and its central part consists of six ATPases that bind to substrate proteins for degradation, unfolding, and translocation into the 20S complex [16]. As a final process, proteins are degraded into polypeptides, which are then chopped into small fragments and ultimately into the single amino acids by the activation of peptidases [17]. The overall system of ubiquitination and proteasomal degradation is known as the ubiquitin–proteasome system (UPS).

Ubiquitination is a reversible and dynamic phenomenon. Deubiquitinating enzymes (DUBs) are a large group of proteases that can partially or completely remove ubiquitin and ubiquitin-like proteins NEDD and SUMO from the target substrate. Currently, 100 DUBs are encoded by the human genome, and they are classified into six different groups: ubiquitin-specific proteases (USPs), ubiquitin COOH-terminal hydrolases (UCHs), Machado–Joseph Domain (Josephin domain)-containing proteins (MJD), the JAB1/MPN/MOV34 family (JAMMs), motif interacting with Ub-containing novel DUB family (MINDYs) [18], and ovarian tumor proteases (OTUs) [19]. DUBs are critical for the regulation of cell survival, immune response [20], and cell differentiation [21,22]; therefore, they are considered as potential drug targets in various diseases.

In the following sections, we would like to emphasize the current understanding of the dysfunction or dysregulation of ubiquitin enzymes and proteasomal degradation in several common diseases. Since the subject is too broad, we try to narrow down the topics by focusing specifically on E1, E2, and E3 enzymes, DUBs, UPS, and their association regarding the progression of cancer, neurodegenerative diseases, immune regulation, and immune-related diseases.

## 3. Ubiquitination-Mediated Regulation in Cancer

Increasing evidence demonstrates the implication of ubiquitin enzymes in carcinogenesis. Although there are plenty of studies about E1 and E2 enzymes in cancer development, most of them focus on E3 ligases. In these studies, E1-activating enzymes are often used for targeting the inhibition of the UPS in cancer treatment [23,24]. On the other hand, E2-conjugated enzymes have been reported in cell cycle stimulation, DNA repair, and the induction of oncogenic signaling pathways during cancer progression via its distinct members: UBE2A, UBE2C, and UBE2D1. Although the overexpression of these E2 enzymes is highly correlated with a poor prognosis of pancreas, lung, breast, skin, and thyroid cancers, several E2 inhibitors have been developed [25]. To date, several compounds were reported to target E2 enzymes as a new class of potential treatment for cancer patients (Table 1). These compounds target the conjugation of E2 enzymes to their substrates [23,24]. 

In contrast to E1 and E2 enzymes, accumulated data exist regarding the role of E3 ligases in cancer. FBW7 (or SCF^FBW7^) is an E3–ubiquitin ligase and a substrate recognition component of SCF, regulating many pro-oncogenic proteins and pathways, such as c-Myc, Cyclin E, mTOR, and Notch [35,36,37]. The phosphorylation of Thr58 and Ser62 residues via FBW7 was shown to be an important regulation for the proteasomal degradation of c-Myc, and the mutation of Thr58 residue causes the tumor progression in Burkitt’s lymphoma [38,39]. Relatively, in chronic myelogenous leukemia (CML), the deletion of FBW7 enhances the expression of c-Myc; it also leads p53-dependent apoptosis in human leukemia-initiating cell (LIC), and eventually, tumorigenesis is inhibited [40]. Moreover, FBW7 inhibited the activity of an oncogenic protein, enhancer of zeste homolog 2 (EZH2); hence, it restrained the migration and invasion of the pancreatic tumor by degrading EZH2 in a FBW7-dependent manner [41]. FBW7 was also associated with the mTOR pathway, in such a way that mTORC2 inhibition induces lipogenesis through the FBW7-mediated degradation of sterol regulatory element-binding protein 1 (SREBP1), which in turn decelerates tumor progression in lung, thyroid, melanoma, and cervical cancer [42]. Additionally, suppressing Notch signaling by FBW7 inhibits the improvement of cancer in adult T-cell leukemia lymphoma (ATLL) [43]. Consistent with the previous observations, the decreased expression level of FBW7 has been demonstrated in other types of cancer, including hepatocellular carcinoma [44], colorectal cancer [45], esophageal squamous cell carcinoma [46], etc. Although many studies strongly support the protective role of FBW7 in cancer, there is still controversy whether targeting FBW7 with a drug promotes or inhibits cancer, since an impairment of FBW7 function in cancer cells was shown to induce chemoresistance by stabilizing oncoproteins [47]. Thus, extensive research is necessary to clarify its drug-resistant role for therapeutic approaches.

MDM2 (Mouse double minute 2 homolog) is another E3 ligase that is involved in cancer. Under normal physiological conditions, the phosphorylation of tumor suppressor protein p53 restrains MDM2–p53 binding and further leads to the overexpression of p53 protein in the cells. In cancer cells, MDM2 polyubiquitinates p53 and cause its proteasomal degradation, which is one of the most frequently altered pathways in human cancers [48,49]. Accordingly, the overexpression of MDM2 has been observed in many cancer types, and thus it has been attracted as a drug target for cancer therapy [50,51].

APC/C, Cdc20, Cdh1, βTrCP, and Skp2 are several E3 ligases that have been suggested to be potential therapeutic molecules in breast cancer. Anaphase Promoting Complex/Cyclosome (APC/C) has two co-activators: Cdc20 and Cdh1. Cdc20 was shown as a prognostic candidate for breast cancer because of its elevated Cdc20 mRNA expression level and its correlation with increased tumor size in cancer patients [52]. Moreover, the inhibition of Cdc20 prevented the migration of breast cancer cell lines and consistently, the overexpression of Cdc20 accelerated the metastatic ability of cancer cells in in vitro conditions [53]. Cdh1 is also dysregulated in breast cancer and melanoma, causing an impairment in genome stability and DNA damage response [54]. The very well-known role of Cdh1 is delaying G1 to S transition in cell cycle regulation via targeting Cdc25A, Skp2, and USP37, which makes it a key protein for cancer development [55]. Similarly, the overexpression of βTrCP (Beta-Transducin Repeats-containing Proteins) was observed in breast and prostate cancer. The underlying molecular basis of βTrCP in breast and prostate cancer is that βTrCP targets the MTSS1 (metastasis suppressor 1) protein and impedes its degradation by UPS, thus promoting tumorigenesis. Therefore, in aggressive breast and prostate cancer, the prevention of MTSS1 degradation is considered as a potential treatment approach for these types of cancer [56]. Furthermore, βTrCP was found to be associated with lung cancer by ubiquitinating its one of the targets, FOXN2 (Forkhead box transcription factor), which plays a role in cell proliferation and radiosensitivity in lung cancer [57] Additionally, it was shown that βTRCP stimulates the ubiquitination and degradation of VEGF (Vascular Endothelial Growth Factor) receptor 2, thereby inhibiting angiogenesis and the migration of papillary thyroid cancer cells [58]. Considering the reported data about βTRCP, its effect in cancer regulation seems to be either positive or negative in a context-dependent manner. Lastly, Skp2 is an oncogene, functioning in the ubiquitination of programmed cell death protein 4 (PDCD4) and inhibiting apoptotic cell death. The enhanced expression of Skp2 was observed in breast and prostate cancer, thereby, therapeutic approaches combining radiotherapy and Skp2 targeting were suggested for breast cancer patients [59]. 

In fact, the data for prostate cancer obtained from various in vitro and in vivo studies imply a scenario in which several E3 ligases are involved in reciprocal interaction between distinct pathways that lead to carcinogenesis. For instance, SPOP (Speckle-type PO2 Protein) E3 ligase promotes ATF2 (Activating Transcription Factor 2) ubiquitination and degradation under normal conditions; however, defective SPOP function induced cell proliferation and invasion [60]. In aggressive prostate cancer, TRIM28 (tripartite motif28) E3 ligase is upregulated and promotes cell proliferation. SPOP was also shown to prevent the interaction of TRIM28–TRIM24 ligases via ubiquitination [61]. E6AP is another important E3 ligase, managing proteasomal degradation of tumor suppressor promyelocytic leukemia protein (PML). The suppression of E6AP results in the reduced growth of prostate cancer cell lines in in vitro conditions, whereas it promote cell senescence in in vivo models. In addition, the knockdown of E6AP was found to make cells more prone to radiation-inducing death [62]. Actually, the underlying mechanism of the tumor-promoting function of E6AP highly depends on its targets. For instance, E6AP targets a cell cycle regulator p27 and inhibits its expression via the E2F1-dependent pathway [63]. On the other hand, E6AP targets the metastasis suppressor NDRG1 (N-Myc Downstream Regulated 1) that was reported in mesenchymal phenotypes of prostate cancer. Pharmacological agents suppressed E6AP-induced cell migration by increasing NDRG1 expression, indicating the E6AP–NDRG1 axis as an appealing target for prostate cancer therapy [64]. 

TRIM proteins are one of the largest subfamilies of E3 ligases directly involve in cancer by controlling cell cycle transition and regulating different oncogenic pathways [65]. Recently, the TRIM7 protein has gained attention in cancer studies, because of its association with Ras, Src, and NF-κB signaling pathways. In hepatocellular carcinoma models, cancer progression was suppressed by TRIM7, which was negatively regulating overactive Src [66]. Besides, a clinical study showed a decrease of Trim7 mRNA expression comparing adjacent normal cells in patients with lung cancer. Thus, the TRIM7 expression has been described as a negative regulation in lung tumors. At the molecular level, TRIM7 promoted apoptosis via the NF-κB signaling pathway and suppressed the proliferation and migration of cancer cells [67]. However, in the Ras-driven lung cancer model, transgenic overexpression of the *Trim7* gene increases the tumor size, while TRIM7 protein deficiency leads to a decrease in tumor growth. The reason was that Ras signaling promoted AP-1 transcription factor activation and stimulated TRIM7 protein that tagged ubiquitin to its co-activator RACO-1 and stabilized it [68]. HUWEI is another E3 ligase that is highly expressed in lung cancer. Inhibition of this E3 ligase activity gave rise to the prevention of cell proliferation and colony formation because of elevated p53 expression [69]. Very recently, the structure and function of HUWE1 and potential drug development targeting the HUWE1–p53 axis were reviewed in a large perspective by Gong et al. [70]. 

One of the most studied E3 ligases, Park2, has been studied in several types of cancer. In hepatocellular carcinoma, defective Park2 function causes abnormal hepatocyte proliferation and leads to avoiding cancer cells from apoptotic cell death [71]. Moreover, the deletion or insufficient expression of Park2 is often described in human glioma, which is correlated with poor survival rates in these patients [72]. 

Recently, deubiquitinases (DUBs) have gained substantial interest as anticancer agents due to their ability to target and catalyze ubiquitin on the substrate proteins. It was reported that the activation or inactivation of specific DUBs, particularly ubiquitin-specific proteases (USP), a major subfamily of DUBs, induced apoptosis in cultured tumor cells [73,74]. Relatively, the overexpression of DUBs was shown in various types of cancer [75]. However, several DUBs were reported as a tumor promoter, such as USP7, USP15, and USP32; some of them were classified as cancer-associated DUBs, such as DUB3, USP19, and USP25 [76,77,78]. Importantly, the coordinative action of DUBs and E3 ligases has also been reported in some cancer types. For instance, USP18 was shown to promote breast cancer growth by activating the Skp2/AKT pathway [79]. 

In breast cancer, the elevated levels of USP37 regulate stemness and cell migration via the hedgehog pathway [58]. Moreover, USP37 induces c-Myc activity by suppressing its degradation, while the inhibition of USP37 increases c-Myc recycling in lung cancer [80]. In contrast, c-Myc inactivation is controlled by the USP28 deubiquitinase [81]. USP7 is commonly found in aggressive brain tumors, leading to p53 stabilization and inhibiting cell death [82]. This mechanism is suppressed by a synthetic drug, 7-chloro-9-oxo-9H-indeno [1,2-b]pyrazine-2,3-dicarbonitrile (HBX 41108). In cancer cells, HBX 41108 treatment increases p53 transcription and recovers p53-mediated cell death. Moreover, USP7 inactivates the ubiquitinated form of Lysine-specific demethylase 1 (LSD1) and promotes cell proliferation through the suppression of cell cycle arrest in brain cancer cells [83]. On the other hand, USP39 influences the function of transcriptional co-activator with PDZ-binding motif (TAZ), which is one of the Hippo tumor suppressor pathway’s proteins. Besides, USP39 inhibits TAZ mRNA expression and promotes tumor growth in glioma [84]. Furthermore, the inhibition of USP14 by a specific inhibitor b-AP15 results in the blockage of cell proliferation through the activation of cell cycle arrest in cancer cells [85,86]. USP14 inhibition also increases the effect of chemotherapeutic agent cisplatin in gastric cancer cells [87]. An in vivo study about the ubiquitin C-terminal hydrolase 5 (UCHL5) and USP14 inhibition by a nickel pyrithione complex displays the suppression of tumor growth in human acute myeloid leukemia [88]. 

Recent studies showed that OTU deubiquitinases can act as either positive or negative regulators in different types of cancer. For instance, OTU deubiquitinase 3 (OTUD3) expression level decreases directly via microRNA-32 targeting, which leads to enhanced proliferation in the HCT116 colorectal cell line [89]. On the other hand, the overexpression of ubiquitin aldehyde binding 1 (OTUB1) was detected in colorectal cancer tissues, which induced the metastasis both in vivo and in vitro studies via triggering the epithelial–mesenchymal transition [90]. In lung cancer, OTUD3 overexpression induced tumorigenesis by stabilizing glucose-regulated protein 78 (GRP78). In addition, Carboxyl terminus of Hsc70-Interacting Protein (CHIP) was found as a negative regulator of OTUD3, and it suppressed the metastasis of lung cancer [91]. Furthermore, the overexpression of OTU domain containing 4 (OTUD4) was revealed to suppress the migration, proliferation, and invasion of cancer cells in breast, liver, and lung via the stimulation of apoptosis by blocking the AKT signaling pathway [92]. 

DUBs are highly specific proteases that act against ubiquitin activity, and their function is crucial for UPS to maintain protein homeostasis. The UPS includes E3 ligases, DUBs, ubiquitin hydrolases, and the proteasome itself. In order to recover proteasomal function, several types of proteasome inhibitors have been investigated, and most of these mainly prevent the excessive degradation of tumor-suppressor proteins. Thiostrepton, dexamethasone, 2-methoxyestradiol, δ-tocotrienol, and quercetin are such inhibitors, which are sufficiently used for the treatment of cancer cells in liver, pancreas, prostate, breast, lung, and melanoma [93]. However, many types of cancer cells are still resistant to those proteasome inhibitors. In line with the current knowledge, there is a consensus on the concept that DUBs generally act as positive regulators in cancer progression. Thus, DUBs may be an ideal candidate for therapeutic approaches in cancer cells, which are resistant to proteasome inhibitors. 

The discussed E3 ubiquitin–protein ligases and DUBs in related cancer types are summarized in Table 2.

## 4. Ubiquitination-Mediated Regulation in Neurodegeneration and Neurodegenerative Diseases

The neuronal accumulation of insoluble proteins is known the major cause of neurodegenerative diseases, including Alzheimer’s disease (AD) [112], Parkinson disease (PD) [113], dementia, progressive supranuclear palsy (PSP), and frontotemporal dementia with parkinsonism linked to chromosome 17 (FTDP-17) [114]. Under normal physiological conditions, UPS, autophagy, or lysosomal degradation systems are responsible for the removal of these accumulated aggregates and misfolded proteins. Today, it is known that some enzymatic mutations in UPS are one of the main reasons for abnormal protein aggregation in neurodegenerative diseases [115].

AD is observed in around 10% of the population over 65 years of age. In the sporadic form of AD, the accumulation of specific neurotoxic proteins, hyperphosphorylated tau, and β-amyloid (Aβ) is the typical characteristic of this disease. E3-ligase Parkin has been shown to have a cytoprotective effect in AD. The study demonstrated that wild-type Parkin expression decreased the intracellular Aβ42 level through UPS and reversed impaired proteasome function [116]. Proteomic analyses showed a wide range of proteins ubiquitinated by Parkin, yet these proteins had no obvious functional association with any cellular mechanism; however, proteins including endocytic trafficking components were over-represented. Further studies are necessary for identifying whether Parkin substrates are functionally related to any known pathway or not [117]. In addition to Parkin, another decreased E3 ligase in AD patients’ brains is “ER related HRD1”. It promotes neural cell survival by mediating the ubiquitination of tau protein. The loss of HRD1 expression results in amyloid precursor protein (APP) accumulation and β-amyloid generation [118]. In addition, it is considered that β-amyloid-dependent oxidative stress leads to the aggresome formation of HRD1, which affects its solubility [119]. The C-terminus of Hsp70-interacting protein (CHIP) is another E3 ligase, and its binding protein Hsp70 is highly expressed in the AD patients’ brain to overcome the phosphorylated tau accumulation by inducing ubiquitination [120,121]. Moreover, the inhibition of CHIP causes an increase in hyperphosphorylated and caspase-3 cleaved tau species [122]. In a recent study, Hsp70 administration is found to be sufficient against late-stage Alzheimer-type neuropathology in a mouse model [123]. Another study showed that the upregulation of CHIP by sulforaphane treatment decreased the accumulation of neurotoxic proteins in a mice model of AD [124]. Therefore, the CHIP/Hsp70 axis seems to be a promising approach for the removal of toxic proteins in the brain. 

In addition to the E3 ligases, DUBs also have critical roles in AD. Ubiquitin C-terminal hydrolase L1 (UCH-L1) was found in tau tangles; the downregulation and considerable oxidative modification of this protein was observed in AD patients. Moreover, the overexpression of UCH-L1 was shown to decrease Aβ plaques and promoted the memory deficiency in an AD mice model [125,126]. Furthermore, OTUB1 (OTU deubiquitinase ubiquitin aldehyde-binding 1) was found in Aβ plaques of AD patients [127], and interestingly, USP14 was able to inhibit tau function [128]. 

In fact, ubiquitination or deubiquitination are not the only defective mechanisms in AD pathology; the impairment of proteasomal function is another main reason for the elevated amount of ubiquitinated protein in the cytoplasm. It is similar to toxic tau accumulation or in other words “tauopathy”, and it is related to decreased proteasomal activity and an increased amount of ubiquitinated proteins in the cell. Within this context, a study has indicated that the activation of cAMP (cyclic Adenosine Monophosphate)–protein kinase A (PKA) by rolipram (phosphodiesterase type 4 (PDE4) inhibitor that increases cAMP levels) leads to the attenuation of proteasome dysfunction by a phosphorylating proteasome subunit [50]. 

The pathology of the second most common neurodegenerative disease PD is characterized by the accumulation of Lewy bodies, α-synuclein, and related multimers in dopaminergic neurons [129]. Parkin/PINK1 is one of the best-studied proteins in this context. Depolarization in the mitochondrial membrane potential activates PINK1 (PTEN-induced putative kinase 1) and stimulates Parkin E3-ligase activity by phosphorylating its Serine 65 residue. Loss-of-function mutations of Parkin/PINK1 were shown to be directly linked to the early onset of PDs [130,131]. Recently, iPSC (induced Pluripotent Stem Cells)-derived midbrain dopamine neurons from PD patients, harboring PINK1 and Parkin mutations, demonstrate an aberrant cytosolic accumulation of α-synuclein and mitochondrial dysfunctions [132]. Additionally, the Parkin/PINK1 pathway is associated with an increased expression of tumor necrosis factor receptor-associated factor 6 (TRAF6) in a PD patient brain [133]. The TRAF6-mediated Lys63 ubiquitination of PINK1 at Lys433 is necessary for mitochondrial regulation and proper PINK1-Parkin-TRAF6 complex formation [134]. In Parkin deficiency, PINK1 triggers mitochondrial antigen presentation in immune cells, which creates inflammatory conditions [135]. Moreover, an idea about the link between the brain and intestinal system is supported by a very recent study of the same research group. Intestinal infection in Pink1 mutant mice shows Parkinson’s-like disease symptoms. Thus, the Parkin/PINK1 axis contributes to an autoimmune response that is directly related to PD etiology [136]. Similar to Parkin/PINK1, recent findings indicate the role of ubiquitination in α-synuclein pathologies. In other words, E3 ligases, namely E6-associated protein (E6AP) and Nedd4, are involved in the α-synuclein degradation process. E6AP has been found to colocalize with α-synuclein, and the overexpression of E6-AP increases α-synuclein degradation in a proteasome-dependent manner [137]. The role of Nedd4 was shown by its overexpression, which causes the hyperubiquitination of α-synuclein in contrast to other ligases. Moreover, the SCF^FBXO7/PARK15^ complex has a considerable ubiquitinase activity, specifically for FBXO7. Glycogen synthase kinase 3β (GSK3β) and Translocase Of Outer Mitochondrial Membrane 20 (TOMM20) have been found as substrates of this complex during PD progression. Furthermore, Teixeria et. al. reported that FBOX7 ubiquitinates and alters GSK3β by Lys63 linkage.

Recent discoveries show the implication of DUBs in PD pathology. For instance, the elevated level of USP13 was found in PD patients [138]. Additionally, UCH-L1 and OTUB1 were shown in Lewy bodies, suggesting that UCH-L1 has the ability to stabilize α-synuclein levels in a context-dependent manner under normal or pathological conditions, while OTUB1 causes amyloid aggregation apart from its DUB activity [127,139]. On the other hand, the activation of UPS via promoting Protein Kinase A phosphorylation eventually stimulated the degradation of α-synuclein [140]. Similarly, the regulation of UPS activity by several chemicals attenuated α-synuclein oligomers [141], suggesting that the recovery of proteasomal deficiency is also critical for synucleinopathies in PD.

The most common polyQ disorders, Huntington’s disease (HD), is characterized by the expansion of CAG repeats in the *huntingtin (htt)* gene as well as the formation of inclusion bodies in striatal neurons [142]. In an in vivo HD model, the genetic inhibition of E3-ligase CHIP showed enhanced neuropathology, indicating that CHIP reduces neurotoxicity by suppressing the accumulation of polyQ [143]. Several other E3 ligases, including UBE3A [144], WWP1 [145], Parkin [146], Hrd1 [147], and HACE1 [148] were also reported to target the Huntingtin protein. 

Recently, one of the DUBs, YOD1, was suggested to decrease proteotoxicity in HD. Neurogenic proteins stimulated YOD1 expression, which caused a reduction in the ubiquitination of abnormal proteins [149]. In addition to ubiquitin enzymes, proteasomal activity is regulated by a member of DUBs, namely USP14. USP14 suppression enhanced proteasome activity and induced the degradation of abnormal proteins such as tau and Ataxin-3 [150]. Similarly, the promotion of proteasomal degradation by USP14 protects neuronal cells from mutant huntingtin-induced cell degeneration [151]. Huntingtin-associated protein (HAP40) is another protein affecting the proteasomal activity in HD. The overexpression of this protein stimulates mutant Htt aggregation due to the impaired UPS [152]. An in vivo study demonstrated that intraneuronal Huntington filaments but not inclusion bodies inhibit proteasome activity [153]. On the other hand, proteasomes are dynamically recruited into inclusion bodies, while these proteasomes remain catalytically active for substrates’ degradation. These results seem to be controversial when compared to previous findings indicating defective proteasomes in HD. Thus, further studies are required for the clear definition of proteasome activity in HD [154]. 

## 5. Ubiquitination in Immune-Related Diseases

Ubiquitination is one of the most essential post-translational modifications, playing an important role in both innate and adaptive immunity. Notably, E3 ligases have a major role in leukocytes activation, differentiation, and development [155]. Besides, DUBs, particularly USPs, are known as an important modulator for T cell function [156]. In accordance, recent studies demonstrated that defective UPS, including aberrant E3 ligases, DUBs, or proteasomes tend to the impairment of immune regulation and thereby cause the development of multiple inflammatory or autoimmune diseases [157]. 

Casitas B-lineage lymphoma (Cbl) proteins, consisting of Cbl-b, Cbl-3, and c-Cbl, are a member of the RING finger-containing E3-ligase superfamily [158]. Due to their RING finger domain, they serve as an E3 ligase to various substrates, forming stable interaction with E2-conjugating enzymes and subsequently promoting ubiquitin transfer from the E2 enzyme to their substrate. In previous studies, Cbls was revealed as a major modulator in immune regulation during the early development of hematopoietic precursor cells into the effector immune cells [159]. *Cb^−/−^* transgenic mice models show that the amount of CD4+ SP thymocytes are significantly elevated by ubiquitination of the CD3 ζ chain of the T-cell receptor (TCR) complex [160]. In B cell development, *Cbl*-deficient B cells show an improvement of BCR (B-cell antigen receptor) signaling and promote several downstream processes, such as Ca^+2^ influx, Syk, and CD79A. Therefore, Cbl is known as a negative regulator of B and T cell development via ubiquitin-dependent degradation [161]. In addition, Cbls are known to regulate the activation of macrophages regarding immune disorders, particularly in cancer and obesity. For instance, *Cbl^−/−^* mice show enhanced tumor growth in colorectal cancer, and the recovery of Cbl expression increases the tumor cell phagocytosis of macrophages through the ubiquitination of surface proteins [162]. In obesity, Cbls act as negative regulators for macrophage activation by suppressing migration signals to adipose tissue. Therefore, insulin resistance and obesity are induced in *Cbl^-/-^* mice. Stimulated macrophage accumulation in adipose tissue results in the secretion of inflammatory cytokines in this model [163,164]. Although there are several studies about Cbls in macrophage regulation, molecular mechanisms underlying inflammatory diseases are still poorly understood.

Cbl-b works with other E3 ligases, which are known as Itch, to prevent the over-reactivity of T-cell-dependent peripheral tolerance. Itch is a monomeric protein, namely HECT (homologous to the E6AP carboxyl terminus), presenting intrinsic catalytic activity. Its main role in immunity is regulating immune cell development and function by mediating protein ubiquitination [155]. Recent studies show that Itch and Cbl-b collaboration leads to proteolysis, independent of ubiquitination of the TCRζ chain. Thus, the signal transduction of the TCR complex is suppressed by preventing ZAP70 and TCRζ chain binding [165]. The cooperative functions of different E3 ligases support the regulation of immune cell homeostasis by ubiquitination [166]. 

Von Hippel-Lindau (VHL) is an E3-ligase complex, consisting of elongin B, elongin C, cullin 2, and ring box protein 1 (RBP1). These ligases are mainly regulated by Hypoxia-inducible factor-1α (HIF-1α), which functions as a critical transcription factor in immune regulation under hypoxic conditions. Under normal conditions, the VHL ligase attaches ubiquitin tags on HIF-1α and prevents target gene activation [167]. In fact, HIF-1α is responsible for the immunosuppressive function of regulatory T cells (Treg) [168]. In VHL deficiency, HIF-1α promotes the elevation of interferon-γ (IFN-γ) production in Treg cells, which causes the emergence of the Th1-like inflammatory phenotype [169], indicating the critical role of the VHL ligase–HIF-1α axis in T cell immunity and differentiation [156]. Low oxygen tension also affects innate immunity via HIF-1α-dependent signaling. An impaired maturation of alveolar macrophages and suppression of neutrophil apoptosis were demonstrated in hypoxia or VHL-deficient conditions [170]. These findings support that the VHL/HIF-1α pathway is responsible not only for T and B cell differentiation but also myeloid cell function during an innate immune response [171]. Moreover, hypoxia and VHL ligases are commonly associated with renal carcinoma. It has been shown that VHL expression is negatively correlated with tumor malignancy through promoting immune responses [172] and in patients, mutated VHL ligase proteins were found to show more natural killer cell toxicity [173]. Taken together, VHL E3 ligases appears to be a remarkable protein for immune regulation and immune-related disease prevention. 

Since ubiquitination is a reversible process, deubiquitinases play a crucial role in maintaining an effective immune response, particularly for constituting adaptive immunity [156]. USPs are the largest family of DUBs including at least 50 members; however, the most important USPs in immune regulation are USP4, USP8, USP9X, USP12, and USP19 [75,174], which are mainly involved in T cell homeostasis as positive or negative regulators. Specifically, USP4 is responsible for promoting Th17 cell differentiation and function. The most established transcription factor RORγt (RAR-related orphan receptor gamma) stabilization for Th17 cell differentiation is mediated by highly expressing USP4. Hence, USP4 was suggested as a potential target for inhibiting Th17-mediated autoimmune disorders, such as rheumatic heart disease [175]. USP8 is another important member of DUBs, which are critical for T cell maturation. The knockdown of T cell-specific USP8 showed that the maturation and proliferation of thymocytes were impaired by a Foxo1–IL-7Rα-dependent mechanism. USP8 mutant mice developed lethal colitis because of impaired T cell homeostasis [176]. Moreover, somatic mutations in the human USP8 gene were suggested to suppress the immune system, which causes the development of Cushing’s Disease. These mutations are associated with the 14-3-3 binding motif (RSYSS) of USP8, which is important for its phosphorylation; thus, mutations in RSYSS motif lead to impairing its DUB activity. In patients with Cushing’s disease, it was observed that adrenocorticotropic hormone (ACTH) is produced at a higher level because of USP8 mutations [177]. Similar to USP8, USP9X acts as a positive mediator for TCR signaling and T cell tolerance. The loss of USP9X leads to the decrease in T cell proliferation and T cell differentiation into helper T cells, hence affecting cytokine production. USP9X deficiency in T cells attenuates TCR signaling and promotes the activation of the NF-κB pathway because of modulating upstream signaling proteins of the NF-κB [178]. USP12 deubiquitinates some of the TCR adaptor proteins, such as LAT (Linker for Activation of T cells) and Trat1, stabilizing the TCR:CD3 complex on the cell surface and consequently preventing the lysosomal degradation of LAT and Trat1 [179]. On the other side, USP19 has a different role in immune regulation compared to other USPs discussed above, since it is related to an innate immune response with its Toll-Like Receptor (TLR)-dependent activity. In a very recent study, Usp19^−/−^ mice treated with poly(I:C) or LPS showed the elevation of pro-inflammatory cytokines and type 1 interferons secretion, indicating that USP19 negatively regulates TLR3/4-mediated signaling by impairing the accession of the essential adaptor protein to TLR3/4 [180].

Cylindromatosis (CYLD) was first discovered as a tumor-suppressor protein [181]. However, it has been shown that CYLD belongs to the deubiquitinating enzyme family, and it removes the Lys63-linked polyubiquitin chain from signaling proteins located upstream of NF-κB, including TNF receptor-associated factor 2 (TRAF2), TRAF6, and NEMO [182]. It is well known that CYLD is a critical factor for T cell development, activation, and function. In fact, the essential role of CYLD in T cell development was identified with threshold activation for the positive selection of T cells via NEMO-dependent NF-κB signaling. In an in vivo study, *Cyld*-deficient mice developed a chronic inflammatory digestive disease, colitis [183]. The underlying mechanism is that CYLD targets TGF-β-activated kinase 1 (TAK1) and inhibits its ubiquitination and autoactivation. This mechanism also activates downstream kinases of TAK1 such as c-Jun N-terminal kinase (JNK) and IκB kinase β (IKKβ) due to the autoactivation of TAK1 [184,185]. In line with this information, the function of CYLD in T cell regulation is considered as a negative feedback mechanism by inhibiting activation of the TAK1 axis of TCR signaling. In transgenic mouse models of CYLD, changes in immune cell function, abnormal hepatocytes, hair, and dental defects were observed [185,186,187]. Furthermore, CYLD polymorphisms were specifically associated with inflammatory bowel disease (IBD) that comprised 2320 patients with Crohn’s disease [188]. 

UPS has been shown as the main modulator for the antigen presentation in immune regulation. More specifically, oligopeptides are hydrolyzed by the proteasome after binding PA28, an activator for hydrolysis, to the ends of 20S proteasome. The process is controlled by a proteolytic cascade including aminopeptidases that are responsible for peptide production for the presentation of MHC-I (Major Histocompatibility Complex-I) [189]. Notably, the MARCH (Membrane-associated RING-CH-type finger) E3 ligase is involved in the reduction of MHC-I surface expression by mediating the polyubiquitination of MHC-I. Therefore, MARCH-mediated MHC presentation is crucial for UPS function in immune regulation [190]. Interestingly, autoantibodies, reacting as self-antigens, were detected in 20S proteasome subunits in several autoimmune diseases, such as systemic lupus erythematosus, primary Sjögren’s syndrome, and myositis. In vitro studies showed that autoantibodies can inhibit proteasome activation by targeting the PA28 binding. On the other hand, proteasome levels in circulation are suggested as an important biomarker for disease progression and potential drug targets, because of their increased levels observed in autoimmune myositis, systemic lupus erythematosus, primary Sjögren’s syndrome, and rheumatoid arthritis [191,192]. 

## 6. Concluding Remarks

**Figure 3:**
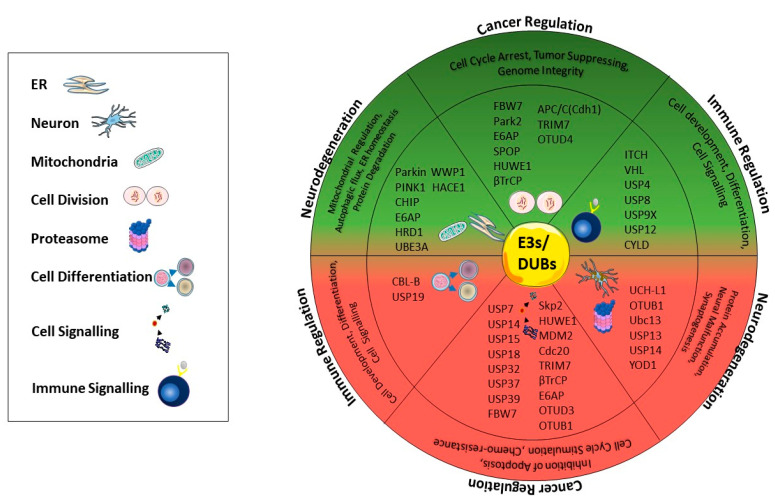
Summary of DUBs and E3 enzymes that play important roles in cancer progression, immune regulation, and neurodegenerative diseases. According to diseases, the enzymes positively or negatively regulate the mechanisms in mitophagy, cell differentiation, cell development, signaling, protein accumulation, neural malfunction, synaptogenesis, inhibition of apoptosis, cell cycle stimulation, mitochondrial regulation, autophagic flux, ER homeostasis, and protein degradation. E3 ligases and DUBs in the upside or downside of the semicircle indicate negative (red area) or positive regulators (green area) of these diseases.

Dysregulation of the ubiquitin–proteasome system including the positive or negative regulation of E3 ligases, DUBs, or proteasomes seriously affects cellular homeostasis and causes the development of serious pathologic conditions, such as tumor suppression or promotion in cancer, protein accumulation in neurodegenerative diseases, and forming an ineffective immune response in the body (Figure 3). Thus, recently a great deal of work has been devoted to the development of novel drugs, targeting proteins that either interfere or inhibit ubiquitination and proteasome activity in disease-dependent manner. Ongoing research studies of a wide range of molecules targeting different ligases show promising data in several pathogenic conditions; nevertheless, the translation of these data into clinical application is still a major challenge. For instance, some compounds are shown to inhibit E3 ligases generally in in vitro conditions, but the effects of such compounds remain unclear in in vivo models [193]. Proteasome-associated DUBs have been suggested as remarkable drug targets due to their lower side effects compared to proteasome-targeted drugs. Currently, DUBs-targeted inhibitors are mainly small molecules, and their development is still in the preclinical stage for inflammatory disease and cancer treatment [194]. The major restriction of DUBs inhibitors is their selectivity, as they may have complex intracellular interactions with several signaling pathways. Proteasome activity is another target in UPS-dependent therapy that became a popular idea to recover prominently in neurodegenerative diseases. Proteasome inhibitors, including bortezomib, carfilzomib, and ixazomib are well-tolerated by patients and therefore approved by the FDA for clinical applications. On the other hand, marizomib and oprozomib are currently under clinical trials [195]. In upcoming years, novel molecules targeting E3 ligases, DUBs, or proteasomes are expected to be validated for therapeutic approaches; thus, a better understanding of the molecular signaling pathways involved in ubiquitination and proteasomal degradation will allow the discovery of novel targeting molecules in cancer, neurodegenerative disease, and immune-related pathological conditions. 

## Figures and Tables

**Table 1:** Summary of compounds targeting E2 enzymes.

Compounds	Targeted E2 Enzyme	References
**CC0651**	Ube2R1	[26]
**Leucettamol A**	Ubc13–Uev1A interaction	[27]
**Manadosterols A and B**	Ubc13–Uev1A interaction	[28]
**BAY 11-7082**	Ubc13	[29]
**UBC12N26**	UBC12–NAE binding	[30]
**Sumoylation Inhibitors**	Ubc9	[31]
**Suramin**	Cdc34–CRL interaction	[32]
**NSC697923**	Ube2N	[29]
**Honokiol**	UbcH8	[33]
**Triazines Compounds**	Rad6B	[34]

Ube2R1: Ubiquitin-Conjugating Enzyme E2, R1, or Cdc34, Ubc13: Ubiquitin-Conjugating Enzyme E2N, UBC12: NEDD8-Conjugating Enzyme, Uev1A: Ubiquitin-conjugating enzyme E2 variant 1A, NAE: NEDD8 activating enzyme, Ubc9: Ubiquitin-Conjugating Enzyme 9, CRL: Cullin-RING E3 ubiquitin ligases, Ube2N: Ubiquitin-Conjugating Enzyme E2N, UbcH8: Ubiquitin Conjugating Enzyme E2 L6, Rad6B: Ubiquitin-conjugating enzyme E2 B or Ube2B.

**Table 2:** Summary of related gene expressions of discussed E3 ubiquitin–protein ligases and deubiquitinases or deubiquitinating enzymes (DUBs) in specific types of cancer.

E3s and DUBs	Disease Association	Related Gene Expression	Ref
**FBW7**	Hepatocellular carcinoma, colorectal cancer, esophageal squamous cell carcinoma	Underexpressed	[44,45]
**MDM2**	Breast cancer	Overexpressed	[50]
**Cdc20**	Breast, pancreatic cancer	Overexpressed	[94]
**APC/C(Cdh1)**	Breast cancer, melanoma	Downregulated	[95,96]
**βTrCP**	Breast, prostate, lung cancer	Expression depends on tissue type	[97,98]
**Skp2**	Breast cancer, prostate cancer	Overexpressed	[99,100]
**SPOP**	Prostate cancer	Downregulated	[101,102]
**E6AP**	Prostate cancer, cervical and lung cancer	Expression depends on tissue type	[103,104]
**TRIM7**	Lung cancer	Expression depends on tissue type	[67,68]
**HUWE1**	Lung, breast, and colorectal carcinoma	Expression depends on tissue type	[105,106,107]
**Park2**	Glioma, hepatocellular carcinoma, and lymphoma	Underexpressed	[71,108]
**USP7**	Prostate cancer, brain tumors	Overexpressed	[78,82]
**USP14**	Gastric cancer	Overexpressed	[87]
**USP15**	Breast Cancer	Overexpressed	[109]
**USP18**	Breast cancer	Overexpressed	[79]
**USP32**	Breast cancer	Overexpressed	[76]
**USP37**	Breast and lung cancer	Overexpressed	[80,110]
**USP39**	Glioma	Overexpressed	[84]
**OTUD3**	Colorectal and lung cancer	Expression depends on tissue type	[89,91,111]
**OTUB1**	Colorectal cancer	Overexpressed	[90]
**OTUD4**	Breast, liver, and lung cancers	Overexpressed	[92]

Fbw7: F-Box/WD Repeat-Containing Protein 7, Mdm2: Mouse double minute 2 homolog, TRIM7: Tripartite motif-containing 7, SPOP: Speckle-type BTB–POZ protein, Cdc20: cell division cycle protein 20, APC/C (Cdh1): Anaphase Promoting Complex or Cyclosome (Cdc20 Homolog 1), HUWE1: HECT, UBA, and WWE Domain Containing E3 Ubiquitin Protein Ligase 1, E6AP: Ubiquitin Protein Ligase E3A, USP: Ubiquitin-Specific Protease.
